# Gaia Therapeutic Apartment - a transdisciplinary response to promote mental illness in adolescence

**DOI:** 10.1192/j.eurpsy.2026.11723

**Published:** 2026-07-31

**Authors:** J. S. Vieira, M. B. Nascimento

**Affiliations:** 1 Instituto de Desenvolvimento e Inclusão Social - IDIS; 2 Pedopsiquiatria da Infância e Adolescência, Unidade Local de Saúde Gaia Espinho, Vila Nova de Gaia, Portugal

## Abstract

**Introduction:**

ApartaMENTE Terapêutico Gaia is a social innovation project that aims to promote adolescents’ mental health, through a transdisciplinary work. The community project is based in Vila Nova de Gaia, Porto, Portugal.

This project is funded by the Portugal Social Innovation initiative, with the support of other social investors, notably the Municipality of Vila Nova de Gaia.

**Objectives:**

- Test and implement a new social innovation response in mental health.
- Reduce negative behaviours associated with the illness in at least 70 young people.
- Improve the subjective well-being of at least 50% of the young people involved in the intervention.
- Validate an intervention methodology through results.

**Methods:**

- Use of a transdisciplinary team composed of a child and adolescent psychiatrist, a psychologist, and a social worker.
- Use of Morenian psychodrama in groups and individually.
- Use of cognitive-behavioural techniques in counselling.
- Use of observation grids and psychometric instruments (EBEPG - General Psychological Well-being Scale (Dupuy, 1984), calibrated for the Portuguese population).

**Results:**

Over the course of nine months, it was possible to take action on 70 young people, 78.6% of whom saw an improvement in their interpersonal well-being ((EBEPG - General Psychological Well-being Scale (Dupuy, 1984)) and 50% register a reduction in negative behaviours resulting from their mental desease.

**Conclusions:**

Public mental health policies for children and adolescents in Portugal are insufficient to provide a adjusted response. Therefore, the emergence of innovative responses supported by public and private funds in the community with validated results are an important tool to scourge of mental illness at earlier ages.

**Disclosure of Interest:**

None Declared

